# Investigation and Computational Analysis of the Sulfotransferase (SOT) Gene Family in Potato (*Solanum tuberosum*): Insights into Sulfur Adjustment for Proper Development and Stimuli Responses

**DOI:** 10.3390/plants10122597

**Published:** 2021-11-26

**Authors:** Sahar Faraji, Parviz Heidari, Hoorieh Amouei, Ertugrul Filiz, Abdullah, Peter Poczai

**Affiliations:** 1 Department of Plant Breeding, Faculty of Crop Science, Sari Agricultural Sciences and Natural Resources University (SANRU), Sari 4818166996, Iran; sahar.faraji@rocketmail.com (S.F.); h.amooei65@gmail.com (H.A.); 2 Faculty of Agriculture, Shahrood University of Technology, Shahrood 3619995161, Iran; 3 Department of Crop and Animal Production, Cilimli Vocational School, Duzce University, 81750 Duzce, Turkey; ertugrulfiliz@gmail.com; 4 Department of Biochemistry, Faculty of Biological Sciences, Quaid-i-Azam University, Islamabad 45320, Pakistan; abd.ullah@bs.qau.edu.pk; 5 Finnish Museum of Natural History, University of Helsinki, P.O. Box 7, 00014 Helsinki, Finland; 6 Faculty of Biological and Environmental Sciences, University of Helsinki, P.O. Box 65, 00065 Helsinki, Finland

**Keywords:** sulfur, sulfotransferase, potato, bioinformatics, protein structure, stimuli coping

## Abstract

Various kinds of primary metabolisms in plants are modulated through sulfate metabolism, and sulfotransferases (SOTs), which are engaged in sulfur metabolism, catalyze sulfonation reactions. In this study, a genome-wide approach was utilized for the recognition and characterization of SOT family genes in the significant nutritional crop potato (*Solanum tuberosum* L.). Twenty-nine putative *StSOT* genes were identified in the potato genome and were mapped onto the nine *S. tuberosum* chromosomes. The protein motifs structure revealed two highly conserved 5′-phosphosulfate-binding (5′ PSB) regions and a 3′-phosphate-binding (3′ PB) motif that are essential for sulfotransferase activities. The protein–protein interaction networks also revealed an interesting interaction between SOTs and other proteins, such as PRTase, APS-kinase, protein phosphatase, and APRs, involved in sulfur compound biosynthesis and the regulation of flavonoid and brassinosteroid metabolic processes. This suggests the importance of sulfotransferases for proper potato growth and development and stress responses. Notably, homology modeling of StSOT proteins and docking analysis of their ligand-binding sites revealed the presence of proline, glycine, serine, and lysine in their active sites. An expression essay of *StSOT* genes via potato RNA-Seq data suggested engagement of these gene family members in plants’ growth and extension and responses to various hormones and biotic or abiotic stimuli. Our predictions may be informative for the functional characterization of the *SOT* genes in potato and other nutritional crops.

## 1. Introduction

The chemical element sulfur (S) is a necessary factor for life found in the amino acid cysteine (Cys) and methionine (Met), certain vitamins (e.g., thiamin and biotin), co-enzymes (e.g., S-adenosyl methionine), iron–sulfur complexes, prosthetic substances, glutathione (GSH) antioxidants, and others natural secondary metabolites [1]. The adequate S in the soil helps plant growth and development, and it is helpful to get a high plant yield of high quality [2]. Moreover, the deficiency of S makes plants susceptible to various biotic and abiotic stresses [3]. An S content ≤ 0.25% in any plant tissue may be considered severe S deficiency; plants with such deficiency have overall chlorosis and yellowish color due to lack of chlorophyll in the early stage of development [4].

Sulfotransferases (SOTs) (EC 2.8.2.-) are sulfate-regulating proteins in various organisms. In plants, the conjugate reaction of sulfate play a vital role in plant growth and development and in response to various stresses [5]. Sulfate is activated by two subsequent steps for the formation of adenosine-5′-phosphosulfate (APS) and 3′-phosphoadenosine-5′-phosphosulfate (PAPS) before being involved in further biochemical reactions [6]. Sulfotransferases (*SOTs*) (EC 2.8.2.-) catalyze the transfer of a sulfate group from PAPS to a hydroxyl group of different substrates [7]. Sulfated substances in plants function as secondary metabolites, hormones in coping with stimulus situations, and use as important S storage substances during the life cycle [8]. Plant SOTs are directly engaged in the sulfation process of desulpho-glucosinolate compounds (ds-Gl), which are important secondary metabolites that provide resistance against multiple biotic/abiotic stimuli in brassicales plants [9]. All SOT proteins can be identified by a histidine residue in their PAPS-binding region and by a specific SOT domain (Pfam: PF00685) [10]. SOT family members are specified by four conserved regions (I to IV) in their protein sequences [11], in which the I and IV regions are highly conserved sections [8]. Three *AtSOT16*, *AtSOT17*, and *AtSOT18* genes in the *Arabidopsis thaliana* (At) genome are responsible for transferring a sulfuryl group to various ds-Gl compounds [8,12]. Various substances, such as brassinosteroids, gibberellic acids, glucosinolates, flavonoids, coumarins, and phenolic acids, can be sulfated by SOT proteins in various plant species [13,14].

Multiple studies indicate that *SOT* genes can regulate plant stimuli responses, stress sensing and signaling mechanisms, and developmental processes. For example, in rice, *Oryza sativa*, expression of some *SOT* gene was observed in root, stigma, and ovary tissues in response to indole acetic acid and Benzyl aminopurine [15]; *BrSOT16* in *Brassica rapa* indicated strong expression in all tissues except for stamen [16]; *ds-Gl AtSOTs*, such as *AtSOT15,* is responsible for circadian control [13]; and expression levels of 11 *OsSOTs* exhibited some up- and downregulation in response to dehydration, high or low temperatures, and hormone stresses in various tissues [15]. Northern blotting of *AtSOT12* revealed that the deduced protein employs flavonoids, brassinosteroids, and salicylic acid compounds as substrates; may be expressed in leaves, flowers, and roots; and responds to abiotic stimuli (such as salt, sorbitol, and cold), hormones, and interactions with biotic pathogens [17,18]. Studies on homologous genes from *B. napus* revealed increased *BNST3* and *BNST4* transcripts during exposure to hormones, low oxygen, xenobiotics, and herbicides [14,19]. This provides evidence for the role of these genes in stress responses and detoxification. Some experimental evidence suggests that SOT may also act as a tyrosyl protein and may involve in phytosulphokines biosynthesis [8]. The glucosinolate and their degradation products provide a defense to plant against insects and fungi. Some evidence shows the role of sulphotransferases in the biosynthesis of glucosinolate. Hence, further exploration of SOT can provide important information for the control of pests [8].

The importance of S during the plant life cycle and associated biological and chemical processes is helpful to overcome S shortage for crop production and improvement. Potato is considered an important food crop after wheat, maize, and rice. Adequate S content in potato plants facilitates the uptake of multiple nutrients, carbohydrate formation, vitamin synthesis, chlorophyll production, seed development and stress, and pest resistance [3,20]. Defective S contents lead to upward curving of potato leaves, along with light-green-to-yellow color. Hence, this leads to poor plant growth, prolate form, and postponed maturity [21]. Previous studies have shown that sufficient S elevated the yield of potato tubers and quality and increased tolerance against various pathogens through the sulfur-induced resistance (SIR) mechanism [3], whereas insufficient S lead to a reduction of several important compounds. [22]. These important aspects necessitate the understanding of plant S biology and adjustment of S nutrition in agricultural programs. Therefore, the identification of important sulfotransferases in the S metabolism may elucidate the S-mediated proper growth and resistance mechanisms in potato. *SOTs* have been identified in *Arabidopsis* (22 members) [8], rice (35 members) [15], and *B. rapa* (56 members) [16]. However, the identification and characterization of SOT proteins in the potato (*Solanum tuberosum*) genome are currently limited. In the current study, various bioinformatics approaches have been utilized to distinguish important cluster *SOTs* and their expression patterns in multiple tissues and during different biotic or abiotic stimuli. Our predictions may assist functional evaluation of the SOT gene family members in potato and related crop species.

## 2. Results

### 2.1. Identification of StSOT Genes

The deduced amino acid sequence of sulfotransferase domain (PF00685) was searched against the Hidden Markov Model (HMM) program and Phytozome database. This led to the identification of 29 putative StSOT proteins; all contained the Sulfotransfer_1 domain and were named according to their chromosomal order (Table 1).

**Figure 1:**
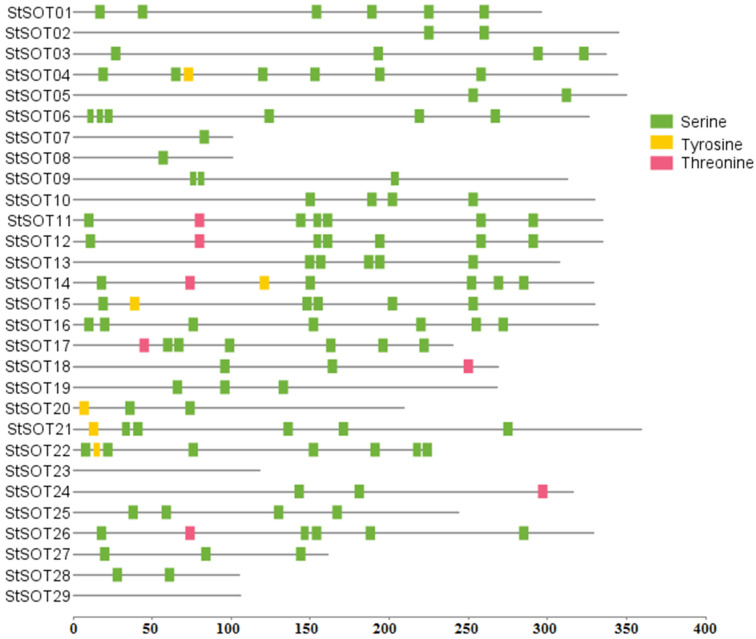
Phosphorylation prediction with scores ≥ 0.95 in StSOT proteins based on serine, threonine, and tyrosine, using NetPhos 3.1 server.

The identified StSOT proteins had diverse lengths, ranging from 101 aa (*StSOT07* and *StSOT08*) to 359 aa (*StSOT21*). Molecular weights (MWs) ranged from 11.83 kDa (*StSOT07*) to 41.56 kDa (*StSOT21*). Most of the identified StSOT proteins (approximately 65.5%) were of acidic nature (theoretical *pI* ≤ 7.0), ranging from 4.95 (cytosolic StSOT28) to 6.83 (cytosolic StSOT13). The subcellular location of proteins indicated that most of StSOTs (approximately 76%) can be considered as cytoplasmic proteins with no putative transmembrane domains (TMDs). StSOT07, StSOT08, and StSOT28 were predicted to be located in the nucleus in addition to the cytoplasm (Table 1). The proteins StSOT01 and StSOT22 were also predicted to be localized in the nucleus and extracellular region. Two StSOT proteins, namely StSOT23 and StSOT29, could also be found in the mitochondria. Not all StSOT proteins contained any putative TMDs in both cytosolic N- and C-terminal regions that can suggest their specific function during the other cellular pathways apart from membrane transport. The StSOT proteins’ post-translational phosphorylation analysis illustrated a wide variety of phosphorylated serine (S) residues, along with some changed threonine (T) and tyrosine (Y) sites (Figure 1 and Supplementary Materials Table S1). The proteins StSOT02, StSOT05, StSOT07, StSOT08, and StSOT28 were predicted to contain a limited amount of phosphorylated regions (in one or two residues) in their amino acid sequences, while some StSOTs, such as StSOT01, StSOT04, StSOT06, StSOT12, StSOT14, StSOT22, and StSOT26, were predicted as the possible highly phosphorylated sulfotransferase proteins in potato.

### 2.2. Phylogenetic Relationships, Conserved Motifs/Residues, and Gene Structure of StSOTs

**Figure 2:**
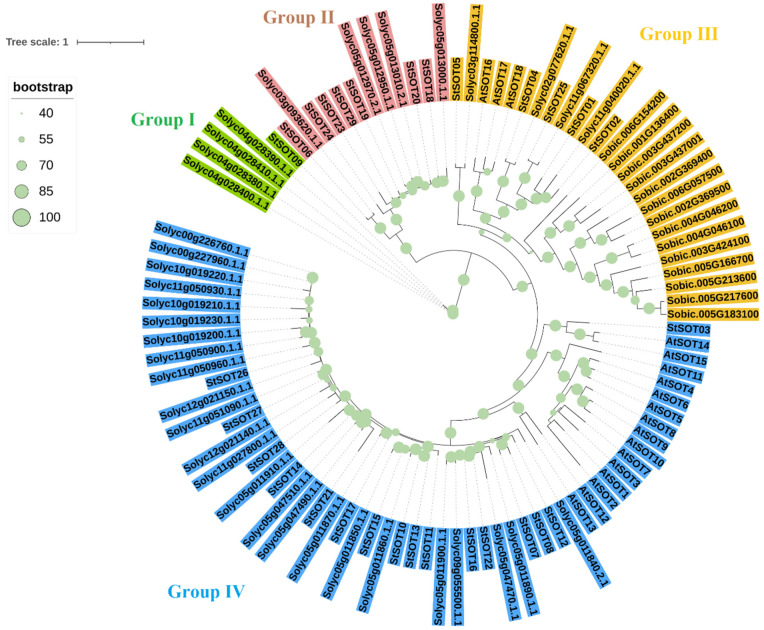
Phylogenetic relationships of SOT proteins from potato, tomato, *Arabidopsis*, and sorghum. The four main clusters were detected based on the ML method in the phylogenetic tree. Abbreviations: St, potato; Solyc, tomato; Sobic, sorghum; At, *Arabidopsis*.

The sulfotransferase proteins from potato, *Arabidopsis*, tomato, and Sorghum were used to generate a phylogenetic tree to classify the SOT proteins into subfamilies (Figure 2). The phylogenetic tree clustered SOTs into the four main groups according to the tree topology and classification of the sulfotransferases in *Arabidopsis*. Four SOTs of tomato along StSOT09 were classified in group I and showed a high genetic distance. Six StSOTs and five SOTs of tomato were located in group II, and all sorghum SOT proteins were grouped with StSOT01, StSOT02, StSOT04, StSOT05, and StSOT25 from potato and AtSOT16, AtSOT17 and AtSOT18 from *Arabidopsis* and four tomato SOTs in group III. Interestingly, all sorghum SOT proteins were separated from dicot SOTs. Group IV was the largest group, and most SOTs of potato, *Arabidopsis*, and tomato were located in this group (Figure 2).

**Figure 3:**
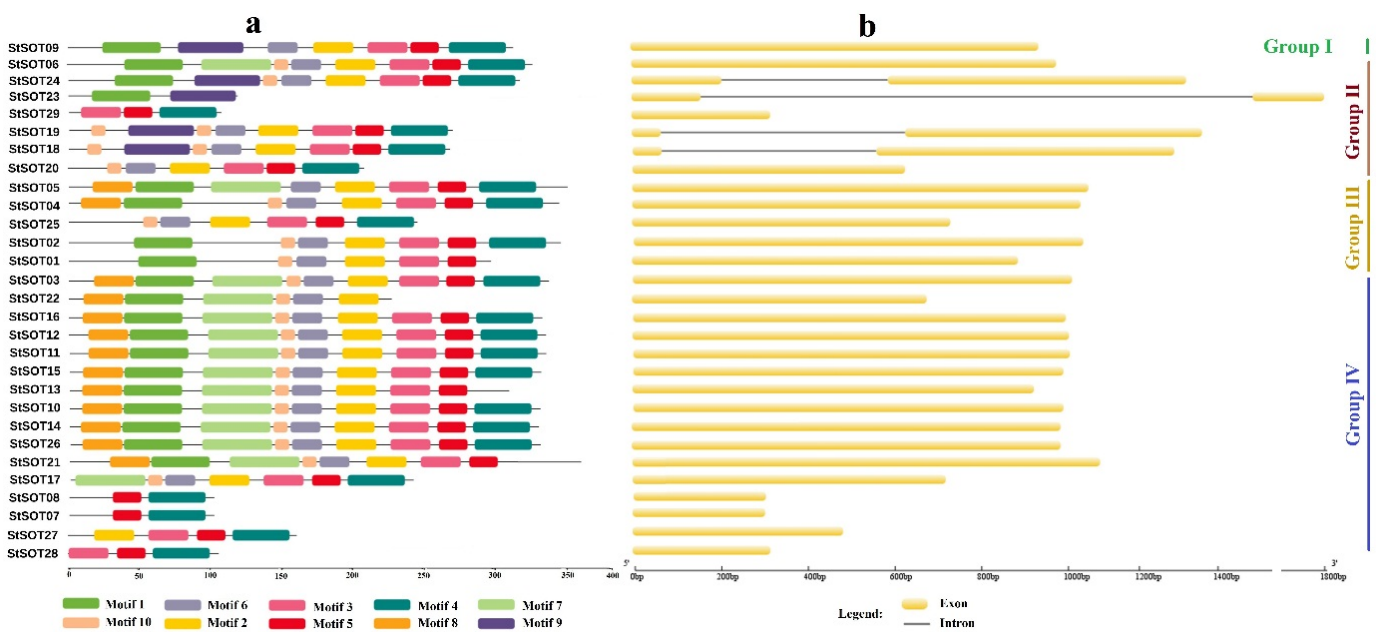
Conserved motifs predicted in the StSOT protein sequences (**a**). Exon–intron structure predicted in the StSOT family genes (**b**). Two important functional 5′ PSB and 3′ PB regions were detected in the motif 1 and motif 6, respectively.

Eight conserved motifs were predicted in the StSOT protein sequences via the MEME program (Figure 3a and Supplementary Materials Table S2). The StSOT proteins belonging to the same phylogenetic group shared an approximately similar conserved motif composition. Five out of the eight predicted motifs, namely motif 1, motif 2, motif 3, motif 4, and motif 6, were identified as having a Sulfotransfer_1 domain (Supplementary Materials Table S2). Motif 1 and motif 6 possessed the critical N-terminal PSB loop and C-terminal PB region, respectively, which are critical for the sulfotransferase activity of SOT proteins (Supplementary Materials Figure S1). The sequences related to these two important motifs are significantly conserved; this high conservation can be found in both cytosolic and membrane sulfotransferases (Supplementary Materials Figure S1).

The N-terminal region 5′ PSB in motif 1 is related to the PSB-loop and helix 3 sections in the sulfotransferase protein structure that encompasses five successive residues engaged in an interaction with the PAPS compound 5′-phosphate region. In this study, the amino acid residues in this motif that are engaged in sulfotransferase catalytic activity include completely conserved Lys-103 and relatively conserved Thr-106 that can be substituted by the functionally similar residues Ser and Cys (Figure 3a and Supplementary Materials Figure S1). Our results revealed that genes within each subfamily have significant similarities in exon and intron numbers. For example, all *StSOT* genes had an intronless structure except for *StSOT18*, *StSOT19*, *StSOT23,* and *StSOT24*, which contained two exons and one intron and were classified into the phylogenetic group II (Figure 3b).

### 2.3. Genomic Distribution, Duplication Assay, and Synteny Relationships of StSOT Genes

**Figure 4:**
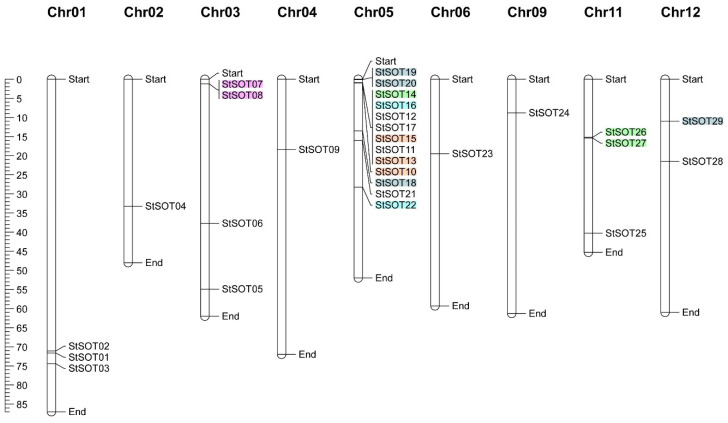
Chromosomal map of StSOT family genes in the potato genome. Five series of duplicated/triplicated *StSOTs* are indicated in different colors. The scale is in mega bases.

All StSOT gene family members were successfully mapped onto 9 out of 12 chromosomes in the potato genome. The chromosomal map revealed an unequal distribution of the gene family members throughout the chromosomes (Figure 4). Chromosome 5 harbored the largest number of *StSOTs* (13 genes), while only one *StSOT* each was predicted to be localized on chromosomes 2, 4, 6, and 9. Nine segmentally duplicated gene pairs categorized into five groups (including duplication and triplication events) were recognized in the StSOT gene family. These groups are indicated with different colors in Figure 4, revealing paralogous pairs. The highest numbers of duplicated/triplicated genes were distributed on chromosome 5, with three duplications and three triplications clustered into the four gene groups (Table 2).

**Figure 5:**
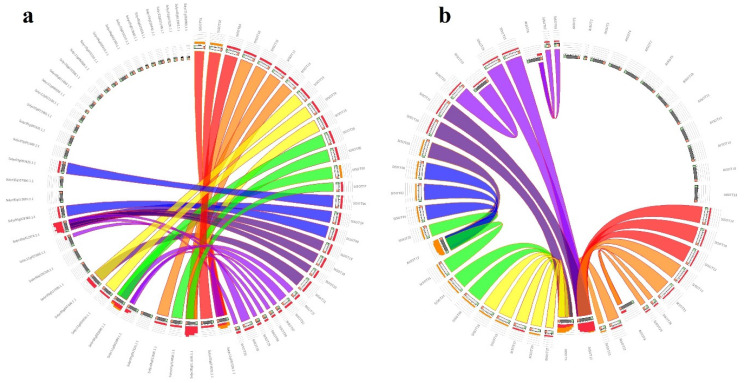
Synteny relationships of *StSOT* genes with orthologs from (**a**) tomato and (**b**) *Arabidopsis*.

Intraspecies synteny results revealed that many of the duplicated blocks were collinear, such as *StSOT07*–*StSOT08* and *StSOT26*–*StSOT27*. The *Ka*/*Ks* magnitudes related to the paralogous pairs ranged from 0.228 to 0.448. According to these ratios, the duplication events were estimated to have occurred between 0.461 to 5.769 million years ago (MYA). The *Ka*/*Ks* ratios < 1 in duplicated gene pairs from StSOT family in potato suggested that these genes have been impressed by purifying selection (Table 2). Synteny analysis has also been performed across the potato and some related plant genomes, which can determine the probable functions of the potato *StSOT* genes (Figure 5). According to the results, all *StSOT* genes showed synteny relationships with their orthologs in the tomato (approximately 35%) and *Arabidopsis* (approximately 32%) genomes. The maximum orthology percentage of the *StSOT* on the potato genome was revealed with tomato. These wide synteny relations at the gene level were considered as confirmation for their close evolutionary relationships. These findings demonstrated the vast rearrangement events of potato chromosomes during the genome evolution process.

### 2.4. Identification of Cis-Regulatory Elements in StSOT Promoters

In the present study, the *StSOT* promoter regions in the potato genome were investigated to identify the putative *cis*-regulatory elements. Several kinds of *cis*-elements for responses to various phytohormones and abiotic stimulus conditions were identified (Supplementary Materials Table S3). The promoter common *cis*-elements, such as the core element TATA-box, CAAT-box, and circadian control element, were identified in all *StSOT* genes. The ABRE (abscisic acid responsiveness), ERE (ethylene responsiveness), and MeJA (Methyl jasmonate responsiveness) factors were predicted as frequently encountered hormone-responding *cis*-elements in most *StSOT* promoters. The light-responsive G-Box and Box 4, wounding-stress-responsive WUN-motif, anaerobic inducible ARE, and stress-responsive MYB elements were identified as the other regulatory *cis*-elements frequently occurring in the *StSOT* promoter areas, suggesting important roles of this gene family in stress responses. The TC-rich repeats (regulating defensive reactions), LTR (low-temperature responsive), TCA-element (salicylic acid-responsive), TGA-element (auxin-responsive), and W-Box (WRKY transcription factors binding region, important for abiotic stimuli responses) were identified as abiotic and hormone-stress-responsive elements predicted in *StSOT08*, *StSOT11*, *StSOT13*, *StSOT16*, *StSOT22*, and *StSOT26*. Multiple regulatory *cis*-elements related to phytohormones and environmental stimuli were identified in most *StSOT* genes, suggesting the critical roles of these genes in potato growth and responses to stress conditions.

### 2.5. Predicted miRNAs for StSOT Genes

**Figure 6:**
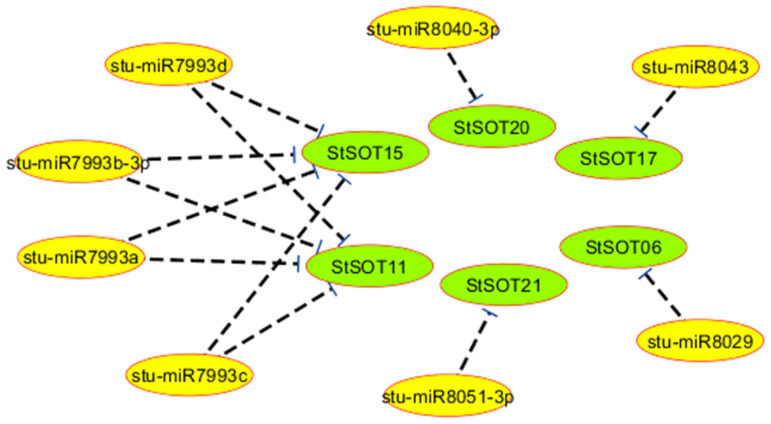
Interaction network between micro-RNAs and *StSOT* genes.

Six *StSOT* transcripts were predicted to be regulated by various miRNAs. For example, the transcripts *StSOT06*, *StSOT17*, *StSOT20*, and *StSOT21* were targeted by stu-miR8029, stu-miR8043, stu-miR8040-3p, and stu-miR8051-3p, respectively (Table 3). Interestingly, four miRNAs, including stu-miR7993a-d, were predicted to target both *StSOT11* and *StSOT15* for inhibition of translation (Table 3 and Figure 6). Furthermore, the targeted regions of *StSOT*s by these miRNAs were predicted into the Sulfotransfer_1 domain region, indicating that the *StSOT* genes are regulated by the identified miRNAs. Remarkably, the identified miRNAs targeted the *StSOT* genes in group IV, illustrating important similarities in their cellular functions during potato growth, development, and degradation. Moreover, targeting of *StSOT* genes by various miRNA isoforms may indicate an important role of these genes during various cellular processes in addition to S assimilation activity.

### 2.6. Protein–Protein Interactions

**Figure 7:**
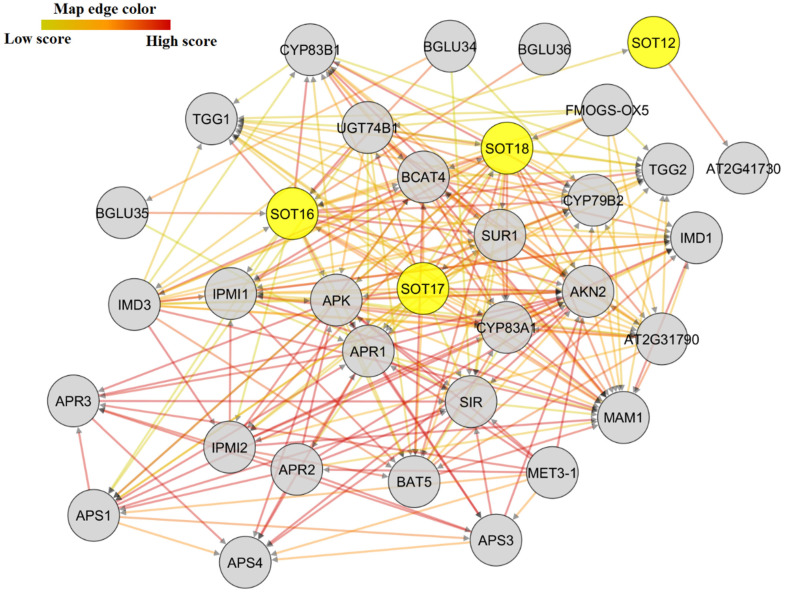
Protein–protein interaction network of SOT proteins, using *Arabidopsis* interactome data through STRING server v11, and improved by using Cytoscape.

The interactome data revealed that SOT proteins interact with proteins involved in transmembrane transport, heme binding, iron–sulfur cluster binding, and transition of phosphate groups (Figure 7 and Supplementary Materials Table S4). SOT16, SOT17, and SOT18, which regulate S compounds and secondary metabolite biosynthetic processes, were likely part of an interaction network with a glucosyltransferase protein that contains transmembrane transporter activity and may respond to stimuli through ion homeostasis. APS (pseudouridine synthase/archaeosine transglycosylase-like family protein), APR (Adenine phosphoribosyl reductase), APK (Adenylyl-sulfate kinase), and MET3-1 precorrin methyl transferase were identified as other transferases working with StSOTs in the biosynthesis of S compounds and secondary metabolites (Supplementary Materials Table S4), which can mediate potato growth and stimuli resistance. The interaction of StSOTs with adenylyl-sulfate kinases can control sulfate assimilation and regulation of S-containing amino acid metabolic processes that are essential for plant reproduction and viability. The APR proteins in the network with StSOTs can adjust iron–sulfur complexes and reduce sulfate for Cys biosynthesis and can be induced by sulfate starvation. The annotation of the SUR, CYP, and AKN proteins that interact with StSOTs revealed the involvement of these interactions in secondary metabolite biosynthetic processes and sulfate assimilation, which modulate plant growth and development and responses to diverse stimuli. The SIR protein was also predicted to be engaged in metal ion transition and secondary metabolite biosynthetic processes that can regulate potato cellular response to stress and sulfate starvation (Supplementary Materials Table S4).

### 2.7. Predicted 3D Modeling, Binding Sites, and Validation of StSOT Proteins

**Figure 8:**
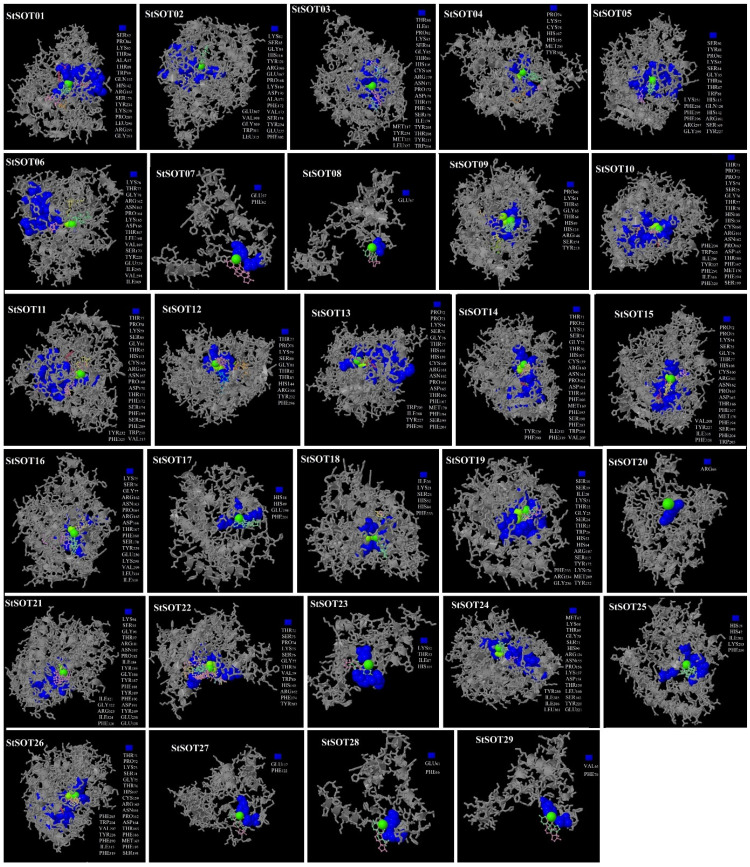
Three-dimensional docking analysis of StSOT protein ligand-binding sites. The binding residues, metallic heterogeneous and non-metallic heterogeneous are shown in blue spacefill, green spacefill, and colorful wireframe, respectively.

The 3D models of StSOT proteins were prepared through the Phyre2 program, under >90% confidence, according to the templates 5mek (as a cytosolic sulfotransferase) and 1q44 and 1fmj (as the P-loop containing PAPS sulfotransferases in *Arabidopsis*). The 3D structure of StSOTs exhibited the conserved typical frames consisting of β3-α8 (as the PSB loop in the proteins 5′ region) and β8-α6 (as the 3′PB motif) (Figure 8 and Supplementary Materials Figure S2). In the model validation, the Ramachandran plot analysis revealed that the qualities of the StSOT protein models varied from 80% to 95%, suggesting the good quality of the predicted 3D models and reliability (Table 4). For further verification, the ProSA server was utilized for evaluation of probable errors within the protein models, indicating the existence of negative z-values in a conformation zone for the predicted models, which can be experimentally distinguished through both X-ray and NMR spectroscopy (Table 4). A remarkable proportion of residues in each protein model was included in the lowest energy regions, indicating decreasing energies in various parts of these putative StSOT proteins.

The highest numbers of protein channels were predicted in StSOT05, StSOT06, StSOT11, StSOT12, StSOT13, StSOT16, StSOT17, StSOT19, StSOT20, and StSOT22, with channel numbers of 11 to 13 (Table 4). Interestingly, some StSOT proteins with considerable similarity in their channel regions, such as StSOT05–StSOT06 and StSOT10–StSOT21, were also included in the same phylogenetic group. Accordingly, this may suggest that the evolutionary divergence of StSOTs can modulate gene characteristics to function in various molecular pathways.

Various numbers of ligand and ligand-binding amino acid residues were identified in the StSOT protein structures (Supplementary Materials Table S5). Some metallic and non-metallic heterogeneous were predicted in the center of the binding region in all candidate protein models (Figure 8). Ser, Pro, Gly, Lys, Tyr, and Arg were predicted as the binding residues in almost all of the ligand-binding regions in the candidate StSOT proteins, which suggest the importance of these residues in positioning on the DNA molecule and in the performance of cellular functions. The Ca, Zn, and Mg ions were identified as the metallic heterogeneous in the StSOT functional domains. Although some binding residues were predicted to be outside of the specific domain, our docking assay indicated that most of these functional regions were included in the Sulfotransfer_1 domain. The binding residues and their metallic or non-metallic interacting heterogeneous revealed that some variations suggest the functional specificity of *StSOT* genes, in addition to their common functions under stimuli exposure and responding to variations in cell metabolism.

### 2.8. Digital Expression Analyses of StSOT Genes

**Figure 9:**
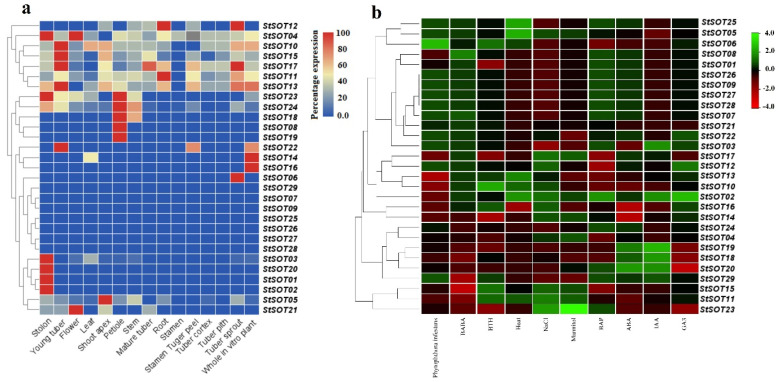
Tissue-specific (**a**) and stimuli-induced gene expression analysis (**b**) of *StSOT* genes in the potato genome based on RNA-Seq data reported by the potato genome sequencing consortium.

The normalized FPKM magnitudes obtained from the RNA-Seq datasets were employed to survey the mRNA transcription patterns of the *StSOT* in various tissues (Figure 9a). All the StSOT family genes were expressed in at least one of the tested potato tissues, except for *StSOT29*, which may play a regulatory role in another cellular pathway. Some *StSOT*s, including *StSOT04*, *StSOT11*, *StSOT12*, *StSOT13*, *StSOT15*, *StSOT17*, and *StSOT24*, exhibited substantial expression levels in all the potato candidate tissues, suggesting the fundamental functions of these sulfotransferases during potato growth and expansion. The developmental functions of these genes may be modulated via the ABRE/ERE-hormones-related and light-responsive Box 4 *cis*-elements present in promoter regions of these genes (Supplementary Materials Table S3). Some of the *StSOT* genes also exhibited a tissue-specific expression pattern. For example, *StSOT09* and *StSOT25* had approximately similar mRNA transcript levels only in the stem and tuber tissues, respectively. The sulfotransferase gene *StSOT27* was strongly expressed in the tuber pith and root tissues, while *StSOT28* had notable FPKM values in the leaf and petiole samples. The other *StSOT*s also had various transcription levels in two, three, or more tissues in potato, suggesting the engagement of these sulfotransferases in a wide variety of cellular functions in these tissues across multiple developmental stages.

The expression patterns of the potato-*SOT*-family-related genes were also examined during exposure to various hormones or biotic or abiotic stresses (Figure 9b). Among the biotic-stimuli-induced *StSOTs*, induction responses were observed under BABA and phytophthora exposures, with notable transcription rates in 19 and 14 *StSOT* genes, respectively (Figure 9b). Eight out of 29 *StSOTs*, including *StSOT10*, *StSOT06*, *StSOT15*, and *StSOT11*, were also upregulated in response to BTH treatment. Amongst the biotic-stress-induced genes, six *StSOT*s, including *StSOT05*, *StSOT06*, *StSOT12*, *StSOT21*, and *StSOT25*, exhibited notable mRNA transcription rates in response to all stimuli, suggesting important roles in defense against pathogens. Thirteen, nine, and seven *StSOT*s were identified as highly expressed genes during exposure to abiotic stimuli NaCl, mannitol, and high temperature, respectively. Of these, *StSOT02*, *StSOT05*, and *StSOT11* exhibited remarkable transcription rates in response to all abiotic stimuli (Figure 9b). In addition, approximately 59%, 55%, 34%, and 24% of the *StSOT*s were substantially upregulated in response to exposure with the BAP, ABA, GA3, and IAA hormones, respectively. Based on our expression assay, *StSOT02* and *StSOT29* can be considered as sulfotransferases responsive to multiple hormones, due to their considerable upregulation when exposed to all the candidate hormones. These transcription levels in different *StSOT*s may be associated with stress-coping *cis*-regulatory elements predicted in the promoter areas. Most of these upregulated *StSOT*s under these stimuli have involvement in biosynthetic processes of secondary metabolites. These predictions may clarify the critical roles of StSOT family-related genes in defensive responses of potato under various stimulus conditions and may identify potential genes for further functional assays to enhance the endurance of potato and related crops to various biotic or abiotic stresses. Although the expression results of RNA-Seq data were not validated by qualitative PCR, several studies showed a high correlation between the results of RNA-Seq and qPCR, for instance in *papain-like cysteine proteases* (*PLCPs*) genes in cotton [23] and rice [24], extensin gene family in tomato [25], GASA gene family in apple [26], *AP2/ERF* genes in wheat [27], and *Aux/IAA* genes in pepper [28]. Moreover, expression patterns of *StSOTs* were compared with their orthologues in *Arabidopsis thalina*, *AtSOTs*, using the eFP Browser database (http://bar.utoronto.ca/efp/cgi-bin/efpWeb.cgi, accessed on 19 November 2021), which showed almost consistent patterns of expression. However, a functional study is needed to describe a perfect conclusion.

## 3. Discussion

The amino acid sequence of the sulfotransferase domain searched against the HMM program and Phytozome database led to the identification of 29 putative StSOT proteins. This revealed extensive variations in physicochemical properties, suggesting an effective role of genomic duplication and integration events during the evolution of this gene family in potato. In the previous studies, 35 *SOT* genes in rice [15], 22 genes in *Arabidopsis* [8], and 56 genes in *Brassica rapa* [16] were identified. It seems that ploidy level and genome size correlate with the gene number in plants [27]. Most of the identified StSOT proteins (approximately 65.5%) were acidic, suggesting a probable correlation of these StSOTs with secretory-pathway-related proteins. The considerable diversity predicted in the *StSOT* gene features may refer to evolutionary changes in the potato genome. Post-translational phosphorylation analysis of StSOT proteins revealed a wide variety of phosphorylated serine residues, along with some changed threonine and tyrosine sites. Some StSOTs, such as StSOT01, StSOT04, StSOT06, StSOT12, StSOT14, StSOT22, and StSOT26, were predicted as putative highly phosphorylated sulfotransferase proteins in potato. Protein phosphorylation can mediate multiple biological processes, such as plant development and stimuli responses [29,30], suggesting the importance of these highly phosphorylated StSOTs during the potato life cycle. Post-translational phosphorylation changes were reported to illustrate the dynamic modulation of plant proteins [31].

According to the conserved motifs predicted in StSOT proteins, the N-terminal region 5′ PSB in motif 1 is related to the PSB-loop and helix 3 sections in the sulfotransferase protein structure. This encompasses five successive residues engaged in an interaction with the PAPS compound 5′-phosphate region [32]. In this study, the amino acid residues in this motif engaged in sulfotransferase catalytic activity include the completely conserved Lys-103 and relatively conserved Thr-106, which can be substituted by the functionally similar residues Ser and Cys (Figure 3 and Supplementary Materials Figure S1). The conserved 3′ PB motif in the C-terminal part of the StSOTs encompassed β-sheet 8 and α-helix 6, which contains Arg-199 and Ser-207 as the interacting sites with the PAPS 3′-phosphate group and modulates its binding selectively [33]. Our results indicated a remarkable structural similarity among these motifs and a fixed number of separating residues in all StSOT proteins, suggesting that *SOT* genes were probably derived from a common ancestral gene. The similarities in the gene structures may also refer to a significant resemblance in expression patterns and regulatory functions in the cell [34]. Moreover, a highly similar distribution of exonic regions may refer to the evolutionary variations that were significantly occurred in the potato genome. The findings suggest that the exon/intron pattern may provide insights into the evolutionary relationships amongst gene family members.

Many *SOT* genes in some plant species may be generated through gene-duplication events [15,16]. At least two whole-genome duplication events have also been reported in the potato genome [35,36], revealing a paleopolyploid origin for this important nutritional crop. Furthermore, the *Ka* and *Ks* rates amongst the duplicated pairs can be considered as an important index to assay the selection pressure and approximate time related to the occurrence of duplications [37]. The *Ka*/*Ks* ratios < 1 in duplicated gene pairs from the StSOT gene family in potato suggest that the genes have been impressed by purifying selection [38]. It was suggested that the genes with conserved functions, pseudogenization, or both may be generated via purifying selection [35]. Regarding the predicted motifs in StSOT proteins, genes within a duplicated gene group might be functionally conserved. This may be attributed to one or more periods of primeval polyploidy occurrence in multiple angiosperm plant lineages [36]. Therefore, these gene duplications in the potato genome may explain the evolutionary novelties observed.

The wide synteny predicted amongst potato–tomato and potato–*Arabidopsis* at the gene level may suggest close evolutionary relationships. The relationships revealed the chromosomal duplication and inversion rearrangement events that organized the *SOT* genes in these genomes [39,40]. Our results suggest that most of the *StSOT* genes share a common ancestor and function with their *SOT* counterparts from tomato and *Arabidopsis*. Despite these close evolutionary relationships between potato and its relatives, some *SOT* genes from *Arabidopsis* and tomato were not mapped on any co-linear blocks compared with potato genes. This may be due to rearrangements and fusions, which can occur extensively on the chromosomes in plants [41,42]. This, in turn, may lead to selective gene loss caused by environmental situations [43]. The information obtained from comparative synteny may further elucidate evolution among crops.

Various stimuli responses are controlled via transcriptional adjustment, which can be modulated by *cis*-elements present in the gene promoter areas [37,44]. According to our results, multiple regulatory *cis*-elements related to phytohormones and environmental stimuli were identified in most *StSOT* genes, indicating the critical role of these genes in potato growth and stress responses. The presence of the light-responsive elements (especially G-Box) suggests that light signals can modulate transcription of *StSOT* genes, and this ultimately regulates genes engaged in defense, such as flavonoid biosynthesis pathways [45,46]. Moreover, miRNAs have also been identified in most organisms and are engaged in various cellular processes, such as stress responses, RNA silencing, protein degradation, and post-transcriptional adjustment [47,48]. Due to the important roles of transcription factors and ion transferases in growth regulation and stress responses in plants, these genes may be important clades of miRNA targets [44,45]. Therefore, the putative miRNAs that targeted six *StSOT* transcripts may mediate post-transcriptional regulation of potato *SOT* genes. Furthermore, miRNAs interact with multiple genes and play an integral role in determining tuberization rates [49]. Remarkably, the identified miRNAs targeted the *StSOT* genes in group IV, suggesting important similarities in their cellular functions during potato growth, development, and degradation. Moreover, targeting of *StSOT* genes by various miRNA isoforms suggests an important role of these genes during various cellular processes in addition to their S assimilation activity [1].

Protein–protein interactions can significantly modulate various cellular functions, such as replication, transcriptional adjustment, growth and development, signaling processes, and coordination of multiple metabolic systems [50,51,52]. The role of StSOT proteins in biosynthetic processes of secondary metabolites indicates their critical functions during proper potato growth and tuberization and stress responses through signaling pathways [50,51]. Moreover, our findings suggest the involvement of some StSOTs in the hormone metabolic processes that are critical for guard cell ABA responses and plant resistance against various herbivores and pathogens. StSOT proteins likely collaborate with proteins from iron–sulfur complexes and amino acid metabolism, which can regulate plant responses to external stimuli [46,50]. Moreover, the collaboration of StSOTs with various development-related proteins can effectively module potato growth and tuberization. As shown in the *StSOT* genes interaction network, APS-kinase, protein phosphatases, ATP-sulfurylase, protein methyltransferase, and NIR can modulate the metabolic pathways of defensive amino acids in potato. The amino acid catabolic system can modulate seedling tolerance against pathogen infection through the overproduction of multiple toxic metabolites, such as serotonin [53]. The construction of these defensive compounds and various S-containing biologically active phytochemicals derived from amino acids, such as tryptophan, is associated with GSH [53]. GSH and tryptophan metabolism may be two essential systems for plant hypersensitive immune responses to various pathogens [53,54]. Furthermore, our interaction network showed that the biosynthesis of amino acid–derived compounds under various stimuli is also regulated through *SOT*-interacting genes, which are necessary for pathogen resistance. Hence, these interacting proteins play indispensable roles during the life cycle of potato cells and sulfotransferases possess a dynamic gene network for metabolism in plants species.

According to the 3D structure of StSOTs, the β-turn and random coil regions in protein structure may provide tolerance to unfavorable circumstances [27,50]. Generally, our predicted 3D models were in good agreement with the parameters related to typical SOT proteins and can be utilized for peptide ligands and as a docking assay. In protein structures, the channels and cavities modulate protein function and can determine their binding specificity [51,55]. The highest numbers of protein channels were predicted in StSOT05, StSOT06, StSOT11, StSOT12, StSOT13, StSOT16, StSOT17, StSOT19, StSOT20, and StSOT22, with 11 to 13 channels (Table 4). The sulfotransferase proteins with similar structures in the channel and cavity regions may also function similarly in cells and under various environmental conditions [27,42,50,51]. Interestingly, some StSOT proteins with considerable similarity in their channel regions (such as StSOT05–StSOT06 and StSOT10–StSOT21) were also included in the same phylogenetic clade. Accordingly, this may suggest that the evolutionary divergence of StSOTs can modulate gene characteristics to function in various molecular pathways. Although some binding residues were predicted outside of the specific domain, according to our docking assay, most of these functional regions were included in the Sulfotransfer_1 domain. The binding residues and their metallic or non-metallic interacting heterogeneous suggest that some variations may possess some functional specificity of *StSOT* genes in addition to their common functions in response to stimuli and variations in cell metabolism [34].

Several studies have elucidated the roles of flavonoid and brassinosteroid metabolites in developmental processes [56]. Flavonoids, usually considered as phytochemical secondary metabolites, and the steroid hormones brassinosteroids, can modulate various physiological processes in the plant. These include growth, enlargement, and immunity via modulation of division, elongation, and differentiation of various cells [57]. Based on promoter site analysis and expression profile of *StSOT* genes, it seems that *StSOT*s are involved in potato growth, development, and response to phytohormones, such as brassinosteroids. The induced mutations and disorders in genes encoding the main building blocks of brassinosteroids and flavonoids disturbed the signaling systems, leading to severe growth failure and impaired organ development, eventually resulting in reduced productivity and yield [57]. The expression levels of *StSOT01*, *StSOT3*, *StSOT21*, *StSOT26*, and *StSOT28* in potato leaf tissue may also be due to multiple light-responsive G-Box and Box 4 *cis*-regulatory elements present in the promoter regions of these sulfotransferases, which can collaborate with flavonoid-producer genes and ultimately regulate the growth process and tuberization in potato [45]. The presence of various hormone-responsive elements in the multiple *StSOT*s may provide further evidence for the importance of these genes in optimal potato optimal growth, development, and tuberization [58]. Further functional investigations of *SOT* genes in potato may lead to enhanced production of some varieties with larger tubers and improved nutritional value.

The transcription levels in different *StSOT*s may be associated with their stress-responsive *cis*-regulatory elements predicted in the promoter regions [59]. Most of these upregulated *StSOT*s under these stimuli indicate involvement in secondary metabolite biosynthetic processes. Secondary metabolites are biologically active and genetically variable compounds found in various plant species that function as natural pesticides and can inhibit insect herbivores [50,51]. The strong defensive responses of *StSOT02*, *StSOT05*, and *StSOT11* during abiotic stress conditions may be related to their regulatory functions in secondary metabolite biosynthetic pathways and salicylic acid signaling [50,51]. Furthermore, potato resistance mechanisms in response to multiple stimuli may be modulated through the interaction and coexpression relationships of sulfotransferases with other stress-responsive genes. These predictions may clarify the critical roles of *StSOT* family-related genes in defensive responses of potato to various stimuli and may identify candidate genes for further functional assays to improve the endurance of potato and related crops to various biotic or abiotic stresses.

## 4. Materials and Methods

### 4.1. Recognition of the StSOT Family Members

The HMM profile related to the SOT domain (PF00685) was first retrieved through the Pfam database [10], and an HMM search (HMMER3.0) was conducted to identify the putative SOT proteins in the potato genome, with an expected value of E-10. The protein HMM profile was also compared to the Phytozome v12.1 database [60] to identify SOT proteins in potato. The recognized non-redundant putative SOT proteins were manually checked for the SOT domain (PF00685) by employing Pfam. The corresponding cDNA and genomic sequences of the distinguished *SOTs* were obtained from Phytozome and genes were named *StSOT01* to *StSOT29*, according to the gene order on the potato chromosomes. In the first, the identified genes were sorted based on their chromosome number, and then the naming for each gene on a chromosome was done randomly.

The physicochemical properties of StSOT proteins, including molecular weights, isoelectric points (pI), and amino acid compositions, were determined with the ProtParam program [61]. Putative transmembrane domains and post-translational phosphorylation changes were predicted in StSOTs, using the SCAMPI program [62] and NetPhos 3.1 server [63], respectively. The location of the StSOT proteins in the cell was also determined with the CELLO program [64].

### 4.2. StSOT Proteins Alignment, Phylogenetic Relationships, and Identification of Conserved Residues

Sequence alignment of StSOT proteins was performed by using the T-COFFEE multiple sequence alignment packages [65]. The phylogenetic relationships were assessed by constructing the maximum likelihood (ML) phylogenetic tree via MEGAX software, according to the protein sequences of SOTs from potato, tomato, Sorghum, and *Arabidopsis*, with 1000 bootstrap replicates [66]. The Multiple Em for Motif Elicitation (MEME) server was also employed to identify conserved protein motifs in StSOT members [67].

### 4.3. StSOT Genes Structure and Chromosomal Map

The exon and intron organizations of potato *StSOT* genes were predicted by using the Gene Structure Display Server [68]. The chromosomal localization of *StSOT* genes was also determined on the 12 chromosomes (Chr) of potato by using the *S. tuberosum* genome info from the Potato Genome Sequencing Consortium database (PGSC) [36]. MapChart software was employed to generate a graphical chromosomal map for *StSOT* genes in the potato genome [69].

### 4.4. Gene Duplication and Synonymous and Non-Synonymous Substitution Rates of StSOTs

The identified *StSOT* genes were evaluated for gene duplication events through the alignment of their cDNA sequences by the ClustalX v.21 program [70]. An identity matrix between the aligned CDSs was prepared, and the duplicated gene pairs were determined as the genes sharing ≥ 90% identity in their nucleotide sequences. The duplicated *StSOT* gene pairs were subjected to codon alignment, using the ClustalW codon alignment tool in MEGAX software. The synonymous (*Ka*) and non-synonymous (*Ks*) substitution values were estimated by utilizing the *Ka*/*Ks* Calculator tool [38]. The time of duplication and divergence (million years ago) were also estimated through a synonymous mutation rate of λ substitutions per synonymous site per year as T= [*Ks*/2λ (λ = 6.5 × 10−9)] × 10^−6^ [71]. The comparative synteny relationships of *SOT* genes among the orthologous pairs between potato and tomato and between potato and *Arabidopsis* at gene levels were visualized through Circos software [72]. A similar method that was introduced for the recognition of *SOT* genes in potato was also used to identify the orthologous genes of other species (tomato and *Arabidopsis*).

### 4.5. Promoter Analysis, miRNA-Targets, and Protein Interaction Assay

The conserved cis-elements existing in the promoter area of *StSOT* genes were predicted by subjecting the 1500 bp upstream region of the start codon ATG in each putative *StSOTs* into the PlantCARE server [73]. The targeting miRNAs for the *StSOT* transcripts were identified by searching the gene-coding sequences against the published miRNAs in the *S. tuberosum* genome in the psRNATarget database [74] and visualized via Cytoscape [75]. The key *StSOTs* in the sulfotransferase family and S compound and secondary metabolites biosynthetic processes were identified according to their gene ontology annotations, and their protein–protein interaction network was predicted via the STRING v11 program [76].

### 4.6. Protein 3D Modeling, Validation, and Docking Analysis of the Ligand Site

The three-dimensional structures of StSOT proteins were predicted through Protein Homology/Analogy Recognition Engine V 2.0 (Phyre2) server [77]. The predicted protein models validation was assessed through Ramachandran Plot Analysis [78] and the Vadar server [79]. Protein secondary structures related to StSOTs were also identified by utilizing Vadar program. The protein molecular voids and pocket/channel numbers were estimated via the BetaCavity Web server [80]. The ProSA server was employed for the calculation of errors and plots in protein structure and validation of the 3D models [81]. Docking analysis of the ligand-binding regions in the predicted protein models was also performed via the 3DLigandSite program [82].

### 4.7. Expression Profiling of StSOT Genes

RNA-Seq data published by the Potato Genome Sequencing Consortium [36] were employed for an expression assay of the *StSOT* genes in multiple tissues and during exposure to various biotic or abiotic stimuli. The biotic stimuli consisted of infection with *Phytophthora infestans*, DL-b-amino-n-butyric acid (BABA), and elicitors’ acibenzolar-S-methyl (BTH) in mixed samples after 24, 36, and 72 h of exposure. The in vitro grown whole plants (after 24 h) were also subjected to three main abiotic stresses, including heat (35 °C), salinity (150 mM NaCl), and drought (mannitol 260 µM). Furthermore, the treatments with four significant hormones, including 6-benzyl amino purine (BAP; 10 µM), abscisic acid (ABA; 50 µM), indole-3-acetic acid (IAA; 10 µM), and gibberellic acid (GA3; 50 µM), were also considered for hormone-stress-induced expression assay of *StSOT* genes. The expression levels of each *StSOT* gene in various tissues and multiple stimuli conditions were identified based on transcripts ID search in the potato genome sequencing consortium RNA-Seq dataset [36], and the transcript magnitudes were determined in fragments per kilobase of exon model per million mapped reads (FPKM) and evaluated by using Cufflinks [83]. Expression levels of *StSOT* genes in tissues were presented based on a percentage. The heatmap related to *StSOT* gene expression was then provided via the Heatmapper program [84].

## 5. Conclusions

Various primary metabolic processes in plants are dependent upon sulfate assimilation. The uptake of inorganic sulfate through sulfate transporters in the plasma membrane of plant cells is the first stage of plant S metabolism. Transportation of S into hydroxyl-containing substrates is the sulfation reaction catalyzed by sulfotransferase genes. *SOT* genes can regulate plant stimuli responses, stress signaling pathways, and developmental processes. The tuberization process in potato can be disturbed by stimuli that disrupt the transportation of photosynthetic products into the tubers, resulting in impaired production. Comprehensive characterization of the SOT gene family using whole-genome sequencing can provide valuable insights into the various developmental and resistance mechanisms and may also identify novel sulfotransferases and their interacting or co-expressed genes. We conclude that StSOTs are diverse proteins, based on their sequence structure and function, and are involved in various pathways related to growth, development, and response to stresses. In the present study, we demonstrated how this important crop effectively employs numerous strategies, such as secondary metabolite biosynthesis, S compound generation, transferase activity, and production of iron–sulfur complexes to modulate various developmental and stimuli resistance processes. Our systematic study of the SOT gene family may provide a better understanding of the function of these genes and insights into their regulatory roles during growth, expansion, and response to stimuli in economically important crop species.

## Figures and Tables

**Table 1:** Identified StSOT gene family members and their characteristics in the potato genome.

Gene ID	Gene Symbol	Protein Length (aa)	MW (KDa)	Isoelectric Point	Subcellular Localization
PGSC0003DMG400000144	*StSOT01*	296	34.38	6.54	Nuclear, Cyt., Extra.
PGSC0003DMG400027779	*StSOT02*	345	40.01	7.12	Cyt.
PGSC0003DMG400003287	*StSOT03*	337	38.80	5.73	Cyt.
PGSC0003DMG400031776	*StSOT04*	344	40.10	5.4	Cyt.
PGSC0003DMG400024622	*StSOT05*	350	40.15	6.54	Cyt.
PGSC0003DMG400018798	*StSOT06*	326	37.56	5.62	Cyt.
PGSC0003DMG400026753	*StSOT07*	101	11.83	5.74	Nuclear, Cyt.
PGSC0003DMG400026752	*StSOT08*	101	11.98	7.68	Nuclear, Cyt.
PGSC0003DMG400039363	*StSOT09*	313	36.15	6.27	Cyt.
PGSC0003DMG400005584	*StSOT10*	330	38.49	6.6	Cyt.
PGSC0003DMG400028349	*StSOT11*	335	39.05	6.8	Cyt.
PGSC0003DMG400028301	*StSOT12*	335	39.17	7.11	Cyt.
PGSC0003DMG400025717	*StSOT13*	308	35.90	6.83	Cyt.
PGSC0003DMG400036271	*StSOT14*	329	38.38	6.42	Cyt.
PGSC0003DMG400046427	*StSOT15*	330	38.58	7.13	Cyt.
PGSC0003DMG400028302	*StSOT16*	332	38.66	6.72	Cyt.
PGSC0003DMG400028350	*StSOT17*	240	28.31	6.31	Cyt.
PGSC0003DMG400015051	*StSOT18*	269	31.41	7.71	Cyt.
PGSC0003DMG400028341	*StSOT19*	268	31.24	7.72	Cyt.
PGSC0003DMG403028340	*StSOT20*	209	24.68	7.67	Cyt.
PGSC0003DMG400002358	*StSOT21*	359	41.56	7.03	Cyt.
PGSC0003DMG400014962	*StSOT22*	226	26.06	6.59	Nuclear, Extra.
PGSC0003DMG400029882	*StSOT23*	118	13.63	6.5	Cyt., Mitochondrial
PGSC0003DMG400020968	*StSOT24*	316	36.90	7.16	Cyt.
PGSC0003DMG400039919	*StSOT25*	244	28.49	5.51	Cyt.
PGSC0003DMG400046295	*StSOT26*	329	38.25	5.83	Cyt.
PGSC0003DMG400046521	*StSOT27*	161	19.20	5.76	Cyt.
PGSC0003DMG400014947	*StSOT28*	105	12.24	4.95	Cyt., Nuclear
PGSC0003DMG400009660	*StSOT29*	106	12.10	8.99	Cyt., Mitochondrial, Nuclear

Cyt., cytoplasm; Extra., extracellular.

**Table 2:** Duplicated gene pairs in the StSOT gene family and *Ka*/*Ks* analysis. Multiple duplication/triplication events were identified in five categories (in different colors in the chromosomal map in Figure 4).

Duplicated Gene Pairs		Duplication Type	*Ka*	*Ks*	*Ka*/*Ks*	Date (Million Years Ago) ^a^
1	*StSOT07-StSOT08*	Segmental	0.0213	0.075	0.284	5.769
2	*StSOT10-StSOT13*	Segmental	0.003	0.006	0.448	0.461
	*StSOT10-StSOT13-StSOT15*		0.010	0.042	0.244	3.230
3	*StSOT26-StSOT27*	Segmental	0.014	0.057	0.254	4.384
	*StSOT14-StSOT26-StSOT27*		0.010	0.033	0.317	2.538
4	*StSOT16-StSOT22*	Segmental	0.015	0.063	0.252	4.846
5	*StSOT19-StSOT20*	Segmental	0.016	0.029	0.544	2.230
	*StSOT18-StSOT19-StSOT20*		0.010	0.045	0.228	3.461
	*StSOT19- StSOT20-StSOT29*		0.006	0.024	0.275	1.846

^a^ Duplication and divergence time (million years ago) were computed based on the T= [*Ks*/2λ (λ = 6.5 × 10^−9^)] × 10^−6^ formula.

**Table 3:** Predicted miRNA-targeted *StSOT* transcripts in the potato genome.

miRNA Accession	Target Gene	miRNA Aligned Fragment	Inhibition Type
stu-miR8029	*StSOT06*	CGAGGUUUUGUUUCUUUUUACCGA	Translation
stu-miR7993a	*StSOT11*	UCAAUUCAAUUGGUGUAUUUUAUA	Translation
stu-miR7993b-3p	*StSOT11*	UCAAUUCAAUUGGUGUAUUUUAUA	Translation
stu-miR7993c	*StSOT11*	UCAAUUCAAUUGGUGUAUUUUAUA	Translation
stu-miR7993d	*StSOT11*	UCAAUUCAAUUGGUGUAUUUUAUA	Translation
stu-miR7993d	*StSOT15*	UCAAUUCAAUUGGUGUAUUUUAUA	Translation
stu-miR7993c	*StSOT15*	UCAAUUCAAUUGGUGUAUUUUAUA	Translation
stu-miR7993a	*StSOT15*	UCAAUUCAAUUGGUGUAUUUUAUA	Translation
stu-miR7993b-3p	*StSOT15*	UCAAUUCAAUUGGUGUAUUUUAUA	Translation
stu-miR8040-3p	*StSOT20*	CUAGUAUUAAUGUUAAUAUUC	Cleavage
stu-miR8043	*StSOT17*	CCGGUUUCAGGUUAAUAUAGU	Cleavage
stu-miR8051-3p	*StSOT21*	UUAUCAUACCAUCUUCUUUAU	Cleavage

**Table 4:** Properties of secondary and tertiary structures of StSOT proteins, validation, and channel numbers.

Protein Name	α-Helixes (%)	β-Sheets (%)	Coils (%)	Turns (%)	Channel Number	Ramachandran Plot (%)	z-Values
StSOT01	132 (44%)	50 (16%)	114 (38%)	76 (25%)	7	93.50%	−8.4
StSOT02	161 (46%)	41 (11%)	143 (41%)	92 (26%)	9	93.90%	−8.73
StSOT03	141 (41%)	50 (14%)	146 (43%)	84 (24%)	8	90.10%	−8.15
StSOT04	148 (43%)	46 (13%)	150 (43%)	88 (25%)	7	93.90%	−8.15
StSOT05	142 (40%)	39 (11%)	169 (48%)	68 (19%)	12	92.80%	−8.61
StSOT06	152 (46%)	39 (11%)	135 (41%)	80 (24%)	12	94.10%	−8.16
StSOT07	47 (46%)	0 (0%)	54 (53%)	32 (31%)	5	90.90%	−1.85
StSOT08	50 (49%)	3 (2%)	48 (47%)	20 (19%)	4	92.90%	−2.01
StSOT09	148 (47%)	44 (14%)	121 (38%)	76 (24%)	10	93.20%	−8.71
StSOT10	151 (45%)	47 (14%)	132 (40%)	72 (21%)	10	94.20%	−8.45
StSOT11	140 (41%)	40 (11%)	155 (46%)	84 (25%)	11	94.00%	−8.52
StSOT12	146 (43%)	42 (12%)	147 (43%)	84 (25%)	12	92.50%	−8.66
StSOT13	120 (38%)	36 (11%)	152 (49%)	96 (31%)	13	81.70%	−7.64
StSOT14	145 (44%)	46 (13%)	138 (41%)	80 (24%)	5	94.50%	−8.6
StSOT15	152 (46%)	50 (15%)	128 (38%)	88 (26%)	3	95.10%	−7.93
StSOT16	148 (44%)	42 (12%)	142 (42%)	76 (22%)	12	91.50%	−9.05
StSOT17	115 (47%)	20 (8%)	105 (43%)	64 (26%)	12	95.40%	−6.17
StSOT18	128 (47%)	30 (11%)	111 (41%)	44 (16%)	7	93.60%	−7.99
StSOT19	132 (49%)	31 (11%)	105 (39%)	64 (23%)	11	95.90%	−7.92
StSOT20	103 (49%)	12 (5%)	94 (44%)	44 (21%)	12	94.20%	−6.67
StSOT21	143 (39%)	43 (11%)	173 (48%)	76 (21%)	10	91.30%	−7.93
StSOT22	94 (41%)	29 (12%)	103 (45%)	72 (31%)	13	79.90%	−5.5
StSOT23	37 (31%)	25 (21%)	56 (47%)	36 (30%)	5	92.20%	−4.01
StSOT24	146 (46%)	35 (11%)	135 (42%)	68 (21%)	9	89.80%	−8.12
StSOT25	113 (46%)	21 (8%)	110 (45%)	60 (24%)	5	93.00%	−5.86
StSOT26	154 (46%)	45 (13%)	130 (39%)	96 (29%)	6	93.30%	−8.77
StSOT27	83 (51%)	3 (1%)	74 (46%)	32 (20%)	4	94.30%	−4.56
StSOT28	49 (46%)	0 (0%)	56 (53%)	24 (22%)	5	80.60%	−3.48
StSOT29	49 (46%)	0 (0%)	57 (53%)	24 (22%)	5	94.20%	−2.78

## Data Availability

Not applicable.

## Supplementary Materials

The following are available online at https://www.mdpi.com/article/10.3390/plants10122597/s1, Figure S1: Multiple sequence alignments of SOT family proteins in potato. The crucial 5′ PSB loop and 3′ PB regions required for sulfotransferase activity are indicated as black rectangles. Figure S2: Predicted 3D of StSOT proteins in potato by using Phyre2 server. Table S1: The identified StSOT gene family members with Sulfotransferase domain (PF00685) from the Solanum tuberosum genome. The proteins post-transcriptional phosphorylation changes have been investigated. Table S2: The conserved motifs predicted in StSOT protein sequences. Table S3: The important cis-regulatory elements predicted in the promoter region of StSOT genes in potato. Table S4: The interaction relationships between Sulfotransferases and the other genes during multiple cellular functions. Table S5: The docking analysis of the Ligand binding site present in StSOT family proteins. The binding residues and metallic and non-metallic heterogenes were detected in blue spacefill, green spacefill, and colorful wireframe, respectively, in the related Figure 8.
