# Supplementary material for: Investigation and Computational Analysis of the Sulfotransferase (SOT) Gene Family in Potato (Solanum tuberosum): Insights into Sulfur Adjustment for Proper Development and Stimuli Responses
Source: Plants (Basel). 2021 Nov 26;10(12):2597. doi: 10.3390/plants10122597 (PMC8707064; doi:10.3390/plants10122597)
Supplement: Supplementary file 1 [file plants-10-02597-s001.zip › Table S1.pdf]

Table S1 The identified *StSOT* gene family members with Sulfotransferase domain (PF00685) from the *Solanum tuberosum* genome. The proteins post-translational phosphorylation changes have been investigated.

Gene symbol	Protein length (aa)	Phosphorylation sites[a]	Score
StSOT01	296	14 S TSRLSENYG<br>41 S PKRESEYPF<br>151 S LLPISILES<br>186 S KAKLSTKVS<br>222 S YWKASVERP<br>257 S DKPLSKEEE	0.986<br>0.979<br>0.978<br>0.991<br>0.992<br>0.998
StSOT02	345	222 S YWKASVEKP<br>257 S DKPFSKEEQ	0.984<br>0.998
StSOT03	337	24 S QETLSEEEL<br>190 S IRPESLGPL<br>291 S KGAISVFEN<br>320 S FERLSNVMD	0.996<br>0.988<br>0.991<br>0.952
StSOT04	344	16 S EEEASSKFK<br>64 S NFKSSWNDI<br>69 Y WNDIYICSA<br>117 S LEFDSPKHS<br>150 S QSLDSTKMI<br>191 S ELLISFEEG<br>255 S GKPFSRDEE	0.961<br>0.991<br>0.979<br>0.991<br>0.981<br>0.995<br>0.986
StSOT05	350	250 S EQPFSLEEQ<br>309 S KNHLSEEMI	0.993<br>0.973
StSOT06	326	14 S IIDSSQSSE<br>16 S DSSQSSEEK<br>17 S SSQSSEEKD<br>121 S SSIYSTKPT<br>216 S YWSESQKRT<br>264 S VWRCSLERL	0.960<br>0.997<br>0.995<br>0.988<br>0.997<br>0.992
StSOT07	101	80 S TVEMSEKLN	0.976
StSOT08	101	54 S NEKLSTGER	0.953
StSOT09	313	78 S IVNRSSKES<br>79 S VNRSSKESL<br>201 S FRKASLEKP	0.996<br>0.997<br>0.992
StSOT10	330	148 S SLPKSIKDS<br>186 S KDIHSIEEI<br>199 S CKGVSPYGP<br>250 S ECPFSIEEE	0.998<br>0.991<br>0.975<br>0.959
StSOT11	335	7 S KSQTSPQIP<br>77 T ILVTTPKSG<br>142 S PRLLSTHMP<br>153 S SLPKSVQDS<br>157 S SVQDSKTKL<br>255 S ECPFSIEEE<br>288 S DGKLSTGEE	0.974<br>0.959<br>0.967<br>0.996<br>0.972<br>0.959<br>0.964
StSOT12	335	8 S SQTSSPTPP<br>77 T ILVTTPKSG<br>153 S SLPKSVQDS<br>157 S SVQDSRTKL<br>191 S IDTNSIEEM<br>255 S EFPFSIEEE<br>288 S NGKLSTGEE	0.991<br>0.959<br>0.997<br>0.969<br>0.981<br>0.972<br>0.995

StSOT13	308	148 S SLPKSVKDS<br>152 S SVKDSKTKL<br>186 S KDIHSIEEI<br>199 S CKGVSPYGP<br>250 S ECPFSIEEE	0.998<br>0.979<br>0.991<br>0.975<br>0.959
StSOT14	329	15 S EDSLSEECK<br>71 T ILVTTPKSG<br>118 Y EHTLYVDGQ<br>147 S SLPKSIMDS<br>249 S ECPFSMEEE<br>266 S LRMCSFENL<br>282 S NGKLSTGEG	0.981<br>0.959<br>0.963<br>0.997<br>0.972<br>0.969<br>0.994
StSOT15	330	16 S QEGLSEECK<br>36 Y WMESYIYNY<br>148 S SLPKSVQDS<br>152 S SVQDSKTKL<br>199 S CKGVSPYGP<br>250 S ECPFSIEEE	0.985<br>0.962<br>0.996<br>0.972<br>0.975<br>0.959
StSOT16	332	7 S TSQNSPPKY<br>17 S QEDQSEECK<br>73 S ILVTSPKSG<br>149 S SLPKSVKNS<br>217 S YWKESIENP<br>252 S ECPFSIEEE<br>269 S LRMCSFENL	0.976<br>0.973<br>0.996<br>0.997<br>0.990<br>0.959<br>0.969
StSOT17	240	42 T SSFTTPRLL<br>58 S SLPKSAQDS<br>62 S SAQDSKTKL<br>96 S KDTNSIEEM<br>160 S ECPFSIKEE<br>193 S TGKLSTGEE<br>219 S TTEMSEKFN	0.984<br>0.995<br>0.954<br>0.983<br>0.971<br>0.991<br>0.972
StSOT18	269	93 S VLLDSIKNS<br>161 S YWEESKKRP<br>247 T KNHMTPKME	0.995<br>0.952<br>0.966
StSOT19	268	63 S EVMHSYSKT<br>93 S ILPNSIKNS<br>130 S LEDLSPLEE	0.990<br>0.975<br>0.995
StSOT20	209	4 Y –MDYYLKTP<br>33 S VLPDSIKNL<br>71 S LEDLSPLEE	0.953<br>0.997<br>0.995
StSOT21	359	10 Y SPPGYNNPT<br>34 S LQDDSLSEE<br>36 S DDSLSEECK<br>133 S VLVPSPERT<br>168 S SLPKSVLDS<br>272 S ECPFSIEEE	0.969<br>0.960<br>0.979<br>0.993<br>0.996<br>0.959
StSOT22	226	8 S SQSSSPKYL<br>11 Y SSPKYLQED<br>17 S QEDLSEERK<br>73 S ILVTSPKSG<br>149 S SLPKSVQNS<br>188 S KDTNSIEEM<br>217 S YWKKSIENS<br>221 S SIENSDKDF	0.995<br>0.961<br>0.993<br>0.996<br>0.973<br>0.983<br>0.994<br>0.997

StSOT23	118	-	-
StSOT24	316	140 S VLPDSFKNS<br>178 S LENPSPLEE<br>294 T KNHMTPKME	0.997<br>0.990<br>0.966
StSOT25	244	35 S INFTSLDDT<br>56 S CLPQSIIDS<br>127 S YRKASKDHH<br>164 S GKPFSKEEE	0.976<br>0.994<br>0.972<br>0.997
StSOT26	329	15 S EDNLSEECK<br>71 T ILVTTPKSG<br>147 S SLPKSVQDS<br>151 S SVQDSKTKL<br>185 S NDTNSIEEM<br>282 S NGKFSTGEA	0.956<br>0.959<br>0.996<br>0.972<br>0.980<br>0.996
StSOT27	161	17 S KDTNSIEEM<br>81 S ECPFSIEEE<br>114 S NGKFSTGEP	0.983<br>0.959<br>0.996
StSOT28	105	25 S ECPFSIEEG<br>58 S NGKFSTGEE	0.970<br>0.996
StSOT29	106	-	-

[a] The phosphorylation regions as the post-transcriptional changes predicted in S: serine , T: threonine and Y: tyrosine residues from StSOT proteins (with the scores higher than 0.950)
