# Supplementary material for: Investigation and Computational Analysis of the Sulfotransferase (SOT) Gene Family in Potato (Solanum tuberosum): Insights into Sulfur Adjustment for Proper Development and Stimuli Responses
Source: Plants (Basel). 2021 Nov 26;10(12):2597. doi: 10.3390/plants10122597 (PMC8707064; doi:10.3390/plants10122597)
Supplement: Supplementary file 1 [file plants-10-02597-s001.zip › Table S2.pdf]

Table S2. The conserved motifs predicted in StSOT protein sequences

Motif	Sites	E-value	Amino acid sequence of motif	Width (aa)	Specific domain/region
Motif 1	18	4.3e-4	NYQGFWTSPRFJQGVIACQQQFQAQDSDIILVTTPKSGTTWL	42	Sulfotransfer_1 (5' PSB)
Motif 2	24	2.1e-4	EEMFELFCKGVSLYGPFWBHVLDYWKQSI	29	Sulfotransfer_1
Motif 3	25	2.0e-3	KYEEJKKDPKIZLKRLAEFLECPFSIEEE	29	Sulfotransfer_1
Motif 4	24	2.5e-5	ENKAFFRKGEVGDWKNYFTIEMSEKLBHITEQKFQGSGLKF	41	Sulfotransfer_1
Motif 5	27	1.5e-3	DEILKMCSFENLSNLEVNKNG	21	-
Motif 6	22	6.2e-3	VYJCRNPKDTFISMWHFTNNLR	22	Sulfotransfer_1 (3' PB)
Motif 7	11	2.2e-3	PIFEQNHPLLVKNPHDLVPFLELTLYVDGQVPBFSSFTSPRLLSTHVPFA	50	-
Motif 8	10	3.2e-1	LQZEGLSEECKKLLSTLPKEKGWMGSYIY	29	-
