# Supplementary figures and images for: Investigation and Computational Analysis of the Sulfotransferase (SOT) Gene Family in Potato (Solanum tuberosum): Insights into Sulfur Adjustment for Proper Development and Stimuli Responses
Source: Plants (Basel). 2021 Nov 26;10(12):2597. doi: 10.3390/plants10122597 (PMC8707064; doi:10.3390/plants10122597)

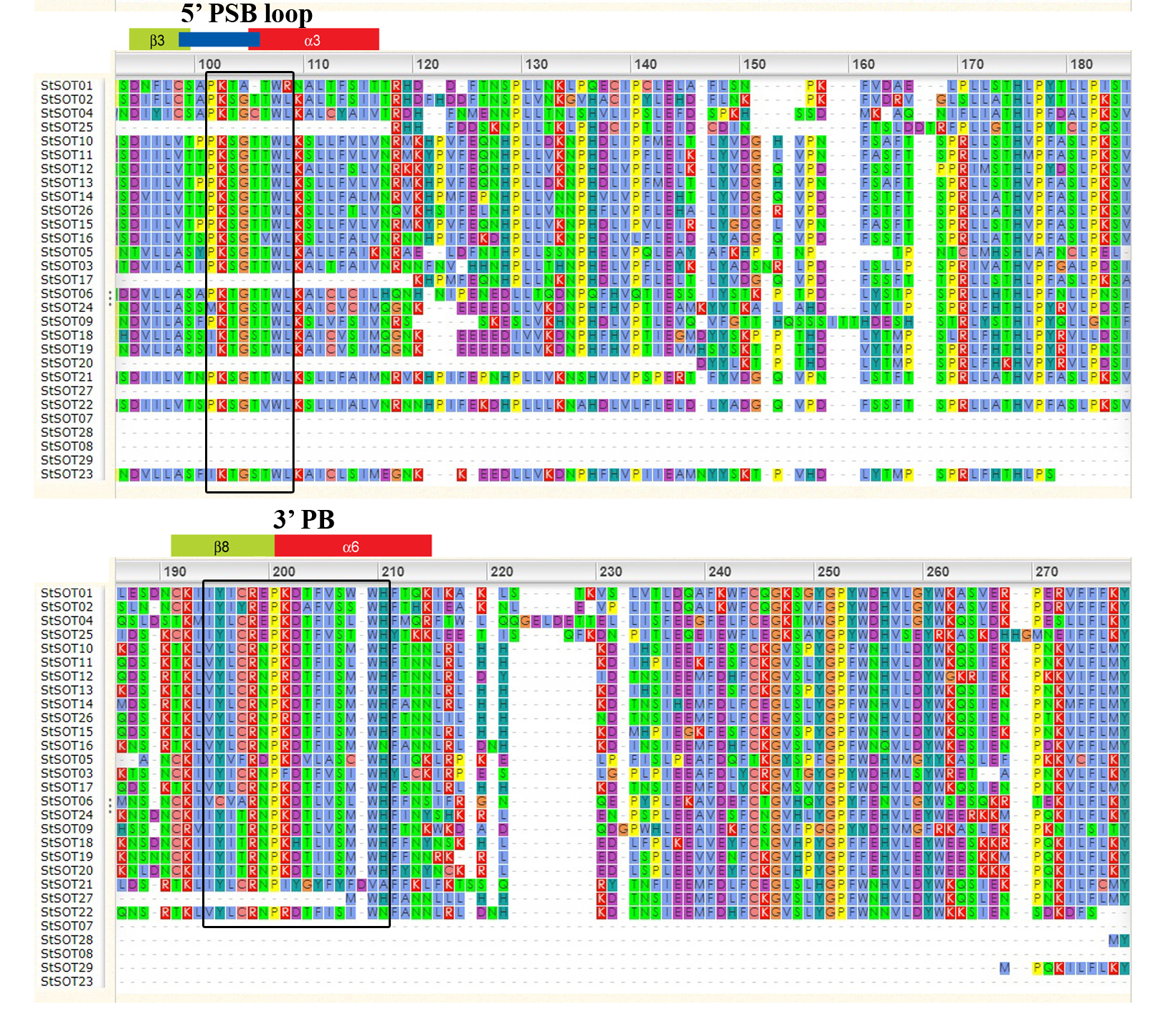

Supplement: Supplementary file 1 [file plants-10-02597-s001.zip › Fig. S1.jpg]

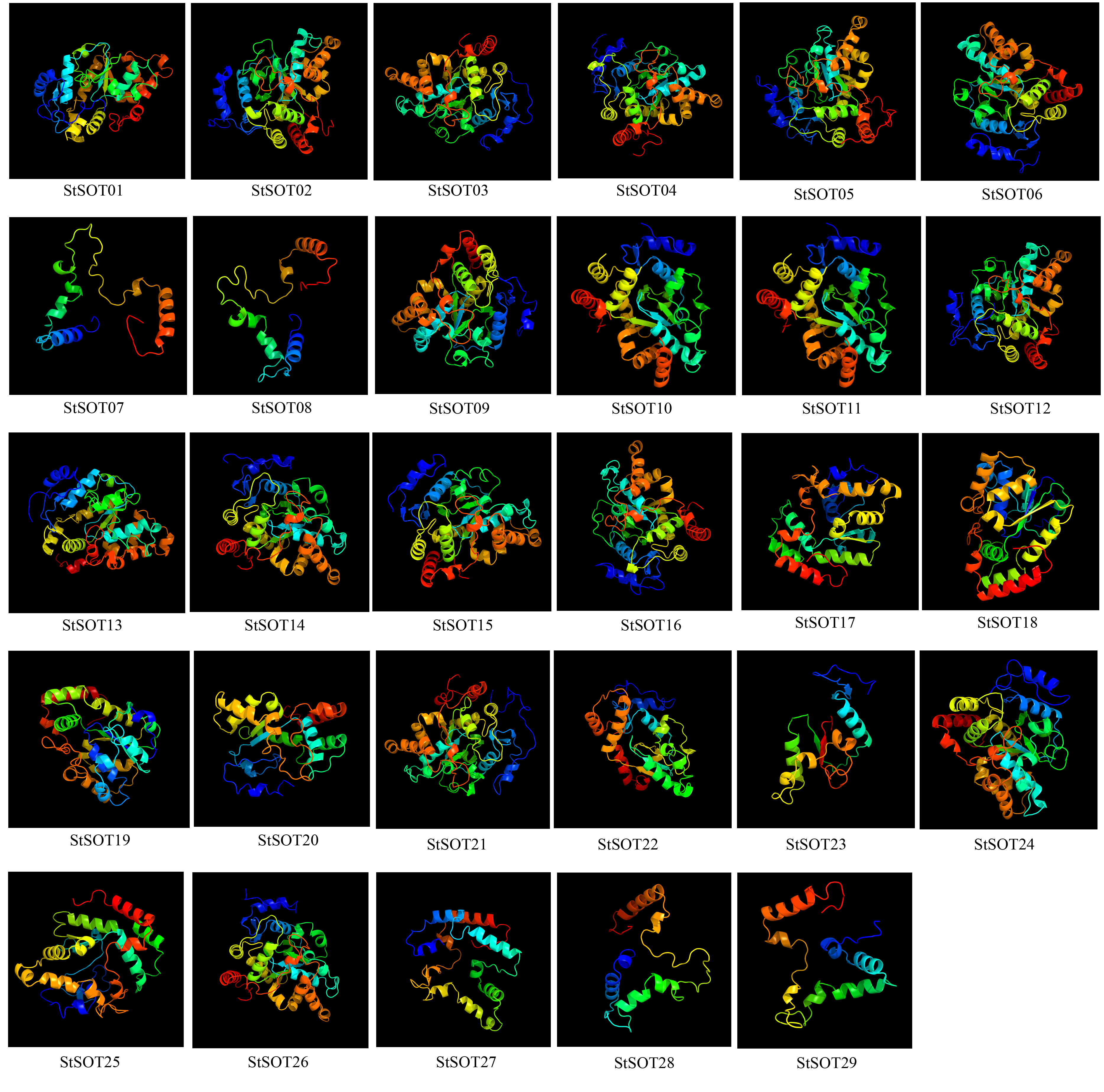

Supplement: Supplementary file 1 [file plants-10-02597-s001.zip › Fig. S2.jpg]
